# Use of financial incentives to increase adult vaccination coverage: A narrative review of lessons learned from COVID-19 and other adult vaccination efforts

**DOI:** 10.1016/j.jvacx.2022.100225

**Published:** 2022-10-06

**Authors:** Nina Schwalbe, Layth Hanbali, Marta C. Nunes, Susanna Lehtimaki

**Affiliations:** a School of Pathology, Faculty of Health Sciences, University of the Witwatersrand, Johannesburg, South Africa; b Spark Street Advisors, New York, NY, United States; c Department of Population and Family Health, Mailman School of Public Health, Columbia University, New York, NY, United States; d Department of Science and Technology/National Research Foundation, South African Research Chair Initiative in Vaccine Preventable Diseases; and South African Medical Research Council, Vaccines & Infectious Diseases Analytics Research Unit, Faculty of Health Sciences, University of the Witwatersrand, Johannesburg, South Africa

**Keywords:** COVID-19, vaccine, vaccination, coverage, hestancy, access, incentive, non-cash, cash, cash tranfer

## Abstract

To encourage COVID-19 vaccination, governments have offered a wide range of incentives to their populations ranging from cash to cows. Often these programs were rolled out at scale before assessing potential effectiveness. To inform future policy, we conducted a narrative review to understand the evidence base informing these programs and the extent to which they are effective. While we found evidence on cash transfers increasing both the coverage and intention to be vaccinated for COVID-19 and other adult vaccines, improvements in coverage were limited. With mixed evidence, lottery programs did not appear to have a consistent meaningful impact on vaccination for COVID-19, and no evidence was identified on the positive effects of other non-cash incentives for COVID-19 or other adult vaccines. We conclude that the impact of cash transfers in incentivizing adult vaccination is marginal and their effectiveness in addressing vaccine hesitancy remains inconclusive.

## Highlights

• Impact of cash transfers in incentivizing adult vaccination is marginal and their effectiveness in addressing vaccine hesitancy is inconclusive.
• Lottery programs do not appear to have a consistent meaningful impact on vaccination.
• Non-cash incentives do not appear to have any positive effects on vaccination.

## Introduction

Access to medicines is often characterized as influenced by four factors: accessibility, availability, affordability, and acceptability [1]. In December 2020, the first coronavirus disease 2019 (COVID-19) vaccine was introduced in the United States (US) through emergency use authorization (EUA) [2]. In 18 months, eleven COVID-19 vaccines are now authorized for use by the World Health Organization (WHO) [3], and more than 66 percent of the world’s population has received at least one vaccine dose [4].

While COVID-19 vaccines are available and affordable in most countries, accessibility is an ongoing challenge in many areas of the world, leading to calls for increased investment in vaccine delivery [5]. In addition, there remain individuals and groups who are “hesitant” towards vaccinations - meaning they display indecision or uncertainty [6], [7]. While this phenomenon has been observed for other vaccine-preventable diseases, it is also true for COVID-19 [7], [8], [9].

The WHO classified vaccine hesitancy as among the ten biggest global health threats in 2019 [10]. Some hesitancy is due to contextual factors, including trust in expertise and authority, or religious and political beliefs. Some is tied to individuals’ concerns about specific vaccines, potential side effects, or even fear of needles. Reasons for hesitancy can also be more rooted in community, geography, or social networks and can be correlated with ethnic or socio-economic background [7], [11]. More recently, hesitancy has been fueled by social media and a so-called “info-demic”. For COVID-19 specifically, a survey in 23 countries found vaccine hesitancy associated with a lack of trust in COVID-19 vaccine safety and science and skepticism about its efficacy [6].

Several factors have demonstrated the potential to shift attitudes and behavior of vaccine-hesitant individuals. These include trust between patients and primary care providers, clarity about vaccine safety and efficacy, honesty around side effects, and an explanation of the role of vaccination in terms of both community and individual protection levels [12]. For childhood immunizations, strategies to overcome parental vaccine hesitancy and strengthen vaccine confidence canter around the key role of primary care in promoting vaccination [13].

While not specific to addressing hesitancy, there is also a wide body of literature that many health care interventions, including vaccines, provider-based “pay for performance” and conditional cash transfers (CCTs), can incentivize care-seeking behavior in both high- and low-income country settings [14].

For COVID-19, many governments and municipalities have put in place incentives aimed at individuals to encourage them to get vaccinated [15]. These included direct cash transfers, lottery tickets, and non-financial incentives, such as doughnuts, blenders, marijuana, herring, or even cows [16], [17], [18]. In New York City, residents were offered a range of items – from a $100 pre-paid debit card, to free amusement park tickets, to a trip to the Statue of Liberty [19], [20].

In this narrative review, we assess the evidence on offering cash transfers and other incentives for increasing adult vaccination uptake. We focus on COVID-19, influenza, hepatitis B, maternal tetanus, and human papillomavirus (HPV). We excluded studies of routine childhood immunizations because the target of those incentives is usually the caregivers rather than the patients themselves.

## Methods

We conducted a narrative review of the literature using MEDLINE, PubMed, and Cochrane databases. The narrative review approach was chosen to enable a thematic approach and inclusion of different types of interventions, outcomes, and populations [21]. The review was limited to peer-reviewed articles published in English and Arabic between 1 January 2012 and 9 February 2022. Relevant articles were identified by conducting an abstract/title search with any of the following key terms: (Incentiv*; cash transfer) AND (COVID-19; SARS-CoV-2; Coronavirus; influenza; flu; maternal/pregnan*/wom*n tetanus) AND (Vaccin* or immunis* or immuniz*). We also hand searched the reference lists of included articles to identify additional relevant publications and searched preprint studies in MedRxiv.

Identified articles were screened by two reviewers independently with the following inclusion criteria: 1) immunization targets the adult population and 2) material or financial incentives are offered. We excluded articles that focused on 1) punitive or negative incentives (e.g., sanctions, restrictions of movement); 2) routine childhood immunization where incentives, when offered, are provided to caregivers rather than patients; 3) incentivizing behavior at the facility level (e.g., a hospital). We also excluded study protocols, opinion papers, and modeling studies. We then synthesized evidence from the articles as described below.

## Results

**Fig. 1:**
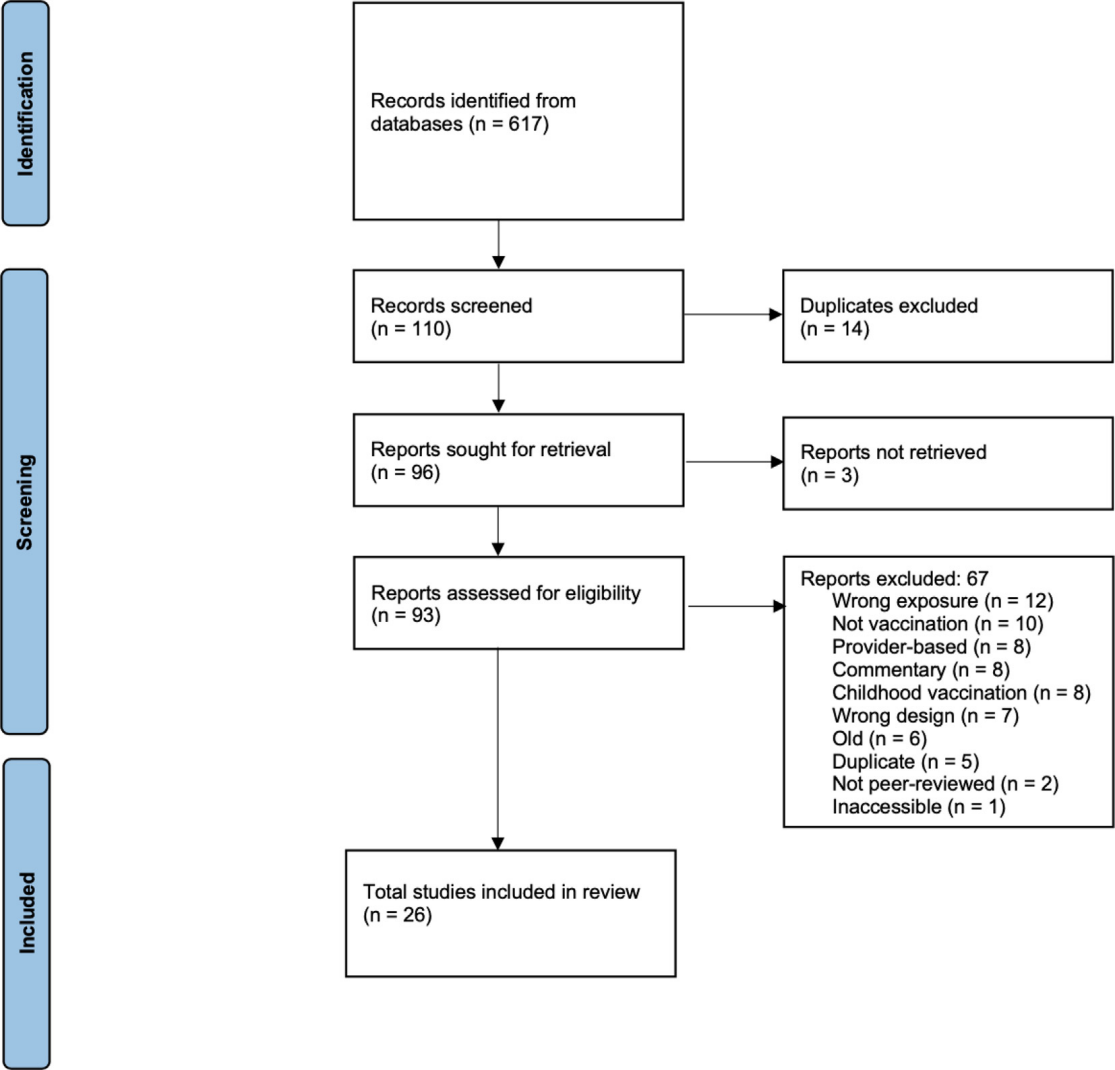
PRISMA flow diagram detailing article selection.

The initial search yielded 617 articles. After title and abstract screening, we conducted a full-text appraisal of 110 articles, excluded duplicates, and identified 26 articles that met our inclusion criteria (Table 1). The rationale for exclusion is detailed in the PRISMA flow diagram in Fig. 1. Three of the articles were systematic reviews. We extracted information on the purpose of the study, key findings as well as a type of incentive, vaccine, target group, and country. We grouped the findings according to whether the incentive was financial or material. The majority of studies were from the United States (US) (13). Studies were also identified from Nigeria (2), Germany (2), and one from each of the United Kingdom (UK), Singapore, Mexico, India, Sweden, and Australia. No studies on COVID-19-related incentives were identified from low- or middle-income countries. While we identified two relevant studies in MedRxiv, we did not include them as they have not yet been peer-reviewed.

**Table 1:** List and summary of included articles.

Reference	Incentive	Vaccine	Target group	Sample size	Country	Findings
Campos-Mercado et al 2021 [24]	Financial incentive	COVID-19	General	8826	Sweden	200 Swedish kroner associated with 4.2 % increase in vaccination rates (baseline 71.6 %)
Kim and Rao 2021 [26]	Financial incentive	COVID-19	General	∼7.5 million (Ecological)	USA	Incentive associated with 44 % increase in first dose, no effect on second dose
Klüver et al 2021 [27]	Financial incentive	COVID-19	General	20 500	Germany	Increased willingness to be vaccinated associated with financial incentive
Robertson et al 2013 [29]	Financial incentive	COVID-19	General	1000	USA	Incentive expected to yield 8 % increase in uptake; most changes in incentive size inconsequential, very large incentive counterproductive
Sprengholz et al 2021 [31]	Financial incentive	COVID-19	General	1349	Germany	Incentives were not associated with a change in participants’ willingness to receive vaccine
Wong et al 2021 [33]	Financial incentive	COVID-19	General	394	USA	Centers participating in incentive associated with higher vaccine uptake
Bronchetti et al 2015 [41]	Financial incentive	Influenza	Students	9358	USA	Incentive associated with an 11 % absolute increase in vaccination uptake (∼doubling of baseline rate)
Clark et al 2021 [43]	Financial incentive	Influenza	Students	66	USA	70 % willing to receive vaccine for $5 or less, half for £1 or less
Yue et al 2020 [45]	Financial incentive	Influenza	Elderly	4000	Singapore	Incentive associated with increased vaccination uptake
Caskey et al 2017 [36]	Financial incentive	HPV	Adolescents	188	USA	Incentive associated with increase in 1st/2nd dose (75 % vs 47 %) and completed course (36 % vs 13 %)
Mantzari et al 2015 [37]	Financial incentive	HPV	Adolescents	1000	UK	Incentive associated with higher uptake of 1st/2nd dose and course completion for previous non-attendees and first-time invitations
Chakrabarti et al 2021 [38]	Financial incentive	Tetanus	General	∼200 000 (Cluster RCT)	India	Increase in maternal tetanus vaccine uptake (79.1 % to 82.8 % in 2006, 84.6 % to 88.9 % in 2016) in conditional cash transfer program
Okoli et al 2014 [39]	Financial incentive	Tetanus	General	20 133	Nigeria	Conditional cash transfer for preventive care associated with 21.66 per 100 000 increase in maternal tetanus vaccine uptake
Sato and Fintan 2020 [40]	Financial incentive	Tetanus	General	2482	Nigeria	Financial incentive associated with large increase in vaccination uptake (85.5 % for 800 naira, 75.7 % for 300 naira, 54.8 % for 5 naira)
Day et al 2016 [34]	Financial incentive	Hepatitis B	People who inject drugs	139	Australia	Incentive associated with higher vaccination completion rate (87 % vs 66 %)
Tressler and Bhandari 2019 [35]	Financial incentive	Hepatitis B	People who inject drugs	Not provided	N/A (Review)	Financial incentives effective at increasing compliance with 3-dose schedule (Odds Ratio 7)
Herrmann et al 2017 [47]	Financial incentive	Multiple	People with substance misuse	5052 (pooled)	N/A (Review)	Incentives effective at increasing completion of 3-dose vaccination course
Salinas-Rodríguez and Manrique-Espinoza 2013 [46]	Financial incentive	Tetanus, pneumococcus, influenza	Elderly	12 146	Mexico	Incentive recipients more likely to receive each vaccination (41–71 % to 46–79 %)
Acharya et al 2021 [22]	Statewide lottery	COVID-19	General	403 714	USA	Positive association between lottery programs and uptake, variable extent of increase
Barber and West 2022 [23]	Statewide lottery	COVID-19	General	∼8 million (Ecological)	USA	Lottery associated with 1.5 % increase in vaccination rates
Dave et al 2021 [25]	Statewide lottery	COVID-19	General	∼258 million (Ecological)	USA	Lottery incentives not associated with statistically significant effect on state vaccination rates
Law et al 2022 [28]	Statewide lottery	COVID-19	General	∼258 million (Ecological)	USA	No significant difference associated with use of lotteries
Sehgal 2021 [30]	Statewide lottery	COVID-19	General	∼8 million (Ecological)	USA	Modest increase (0.98 %) in vaccination rates associated with lottery state
Walkey et al 2021 [32]	Statewide lottery	COVID-19	General	∼258 million (Ecological)	USA	No significant difference in vaccination uptake associated with introduction of lottery incentive
Cheema et al 2013 [42]	Time off	Influenza	Health and social care workers	154	USA	Quarter of respondents reported one-hour time-off incentive influenced vaccination decision
Lytras et al 2016 [44]	Mix of incentives	Influenza	Health and social care workers	Not provided	N/A (Systematic Review)	No significant difference associated with individual and group incentives (gifts, raffle, free drinks, bonus for meeting target)

## Incentives

We identified a total of 26 articles that discussed incentives and vaccination, including vaccines for SARS-CoV-2 (12 studies) [22], [23], [24], [25], [26], [27], [28], [29], [30], [31], [32], [33], hepatitis B (2 studies) [34], [35], HPV (2) [36], [3], maternal tetanus (3) [38], [39], [40], influenza (5) [41], [42], [43], adult tetanus, pneumococcus, and influenza (1) [46], and a mix of different vaccinations (1) [47]. Target groups, if specified, included adolescents (2), students (2), elderly (2), health and social care workers (2), people with substance misuse disorders (1), and people who inject drugs (2). Of these, 18 were about financial incentives, five about lotteries (or an opportunity to win a cash prize), and one about time off. Two were about a mix of different incentives. We found some evidence that financial incentives may increase immunization coverage in the short term, while material incentives did not have an impact on uptake.

## Hepatitis B, HPV, Influenza, and maternal tetanus

### Conditional cash transfers

We identified twelve studies, including two systematic reviews, on conditional cash transfers for incentivizing uptake of hepatitis B, HPV, influenza, or maternal tetanus vaccinations and found evidence that cash transfers (or the promise thereof) can increase vaccination uptake or stated intent to be vaccinated.

A study by Yue et al. in Singapore randomly sampled 4,000 people aged 65 and older to take part in an experimental design where some participants were offered a partial subsidy (shopping vouchers worth 10/20/30 SGD (approx. 7.5/15/23 USD) following receipt of a seasonal influenza vaccine which they paid for themselves) [45]. Increasing incentive from 10 to 20 SGD was associated with improved vaccine uptake, while no additional gains were found with a further increase to 30 SGD. Non-working people were more sensitive to the amount offered than those working, being over 2.3–2.8 times more likely to get vaccinated if incentivized [45].

Clark et al. assessed incentives required to promote influenza vaccine uptake among 66 college students in Pennsylvania, USA [43]. Among students that had previously been offered a vaccine but had not yet been vaccinated, 85 % of participants responded that they were willing to receive the influenza vaccine for 20 USD or less, 70 % for 5 USD or less, and half required less than 1 USD. All participants in the study were willing to accept a vaccine for a certain price, and for the overwhelming majority, this was less than 100 USD. Participants were not asked if they would take the vaccine without remuneration [43].

Bronchetti et al. also focused on influenza vaccines for college students, investigating the effect of social networks and financial incentives [41]. They found that a financial incentive (30 USD) offered within two weeks of vaccine uptake was effective at increasing vaccine uptake among college students, with an 11 % difference between the study and control group [41].

Two systematic reviews have been conducted on the use of incentives to increase uptake of hepatitis B vaccine among people who inject drugs, both of which concluded that financial incentives are effective at increasing coverage. Herrmann et al., based on 5 randomized control trials (RCTs) and 1 historical trial, reported incentives being associated with an absolute increase of between 21 % and 36 % in vaccine uptake compared to control [47]. Tressler and Bhandari, based on 3 RCTs, found that the pooled incentive group had seven times the odds of completing a 3-dose vaccine course compared to control [35].

Mantzari et al. investigated the effects of financial incentives on HPV vaccine uptake in the UK. Girls 16–18 years old were offered vaccination (control) or vaccination with a voucher upon completion of the three-dose vaccination course (treatment) [37]. Incentives were associated with increased uptake of the first dose and completion of the course among both those invited for the first time and those who had been previously invited for vaccination but not attended. (Among those invited for the first time, OR 1.63 for receiving the first dose in the treatment group compared to control, OR 2.15 for completing the course. Among those with the previous non-attendance, OR 2.65 for the first dose, OR 4.28 for completing the course.) However, the uptake in the treatment group was still below the national target, suggesting that the incentive may have been more effective at reaching the undecided than the hesitant [36]. A randomized control trial by Caskey et al. found that 36 % of adolescents receiving incentives completed the full HPV vaccination course and 75 % received one or two doses, compared to 13 % completing the course and 47 % receiving one or two doses in the control group [36]. The offer of an incentive for HPV did not result in a difference between the experimental and control groups vis-à-vis seeking influenza vaccination at a later date [36].

In one of four studies identified from a low- or middle-income country, Salinas-Rodríguez and Manrique-Espinoza assessed the effect of cash transfer on tetanus, pneumococcal, and influenza vaccine uptake among the elderly in the 731 poorest rural communities in 13 Mexican states using a cross-sectional design matching cases to controls [46]. 44 000 households were surveyed, and vaccination status was self-reported. Those incentivized were more likely to have each of the three vaccinations (influenza 46 % vs 41 %; pneumococcal 52 % vs 45 %; tetanus 79 % vs 71 %) [46]. The amount of the cash transfer was not reported nor was there any analysis of cost-effectiveness more broadly.

Three studies looked at the use of conditional cash transfers on the uptake of maternal tetanus vaccines. A pilot program in Nigeria, where the incentive was part of a broad range of interventions from antenatal to postnatal care, demonstrated a significant increase of 21.66 vaccinations per 100 000 catchment population per month over the baseline (9.23 to 34.08) [39]. Another also from Nigeria, where a payment was made at the clinic upon receipt of the vaccine, showed a positive effect of a financial incentive when the incentive was large enough to compensate for the cost of travel to the clinic [40]. A study of a program in India, also offering an incentive for a range of maternal health interventions, including tetanus vaccine during antenatal care visits, similarly demonstrated a small positive effect on uptake [38].

### Non-cash transfers

Evidence of non-cash incentives for hepatitis B, HPV, influenza, or maternal tetanus vaccinations was limited. We found only one systematic review and one cross-sectional study, both focusing on seasonal influenza vaccinations for health care workers. According to these studies, non-cash incentivization did not increase vaccine uptake. A systematic review by Lytras et al. (11 studies in total) on interventions to increase seasonal influenza vaccine uptake in health care workers found that incentives (including gifts, perks, raffles at the individual level or free drinks, bonus/rewards for meeting target at the group level) did not significantly affect influenza vaccination uptake [44]. Cheema et al. surveyed health care workers on the effect of a one-hour time off incentive for influenza vaccination and found no association with this influencing their decision to get vaccinated [42].

## Covid-19

### Conditional cash transfers

We identified six studies on COVID-19 vaccination, four of which focused on ‘intent to vaccinate’ with a hypothetical offer of a vaccine and two assessing actual cash transfer programs. The studies reported a positive effect of financial incentivization, with an increase in uptake ranging from 4.2 % to 9 %. The effect was more significant among those that identified themselves as “undecided” than those who “refused.” One study found a significant increase in first-dose uptake but no difference in course completion [26].

A randomized survey conducted prior to the availability of vaccines by Robertson et al. in the USA of one thousand American adults in December 2020 on the intent to vaccinate for COVID-19 demonstrated financial incentives would yield an 8 % increase in uptake [29]. While the incentives proposed in this study were dramatically larger than in other studies (1000, 1500, or 2000 USD), the size of the incentive did not significantly affect the outcome. The middle-income group was most responsive to an incentive. For Black and Latino respondents, the largest incentive (2000 USD) was counter-productive [29].

A study in Germany in November 2020, also prior to the availability of vaccines, asked randomly selected participants (non-probabilistic sample; quota representative) about intent to vaccinate for COVID-19, and did not find any effect of a potential financial incentive, even after controlling for participant financial status [31]. A later nationally representative survey conducted in March 2021, also on intent to vaccinate, showed that financial incentives would have an impact and that doubling the incentive from 25 Euros to 50 Euros corresponded to a doubling effect on vaccine uptake [27]. However, the effect size was noted only for those who were declared as “undecided” (at 5 percentage points). Those who “refused” were less likely to respond to the incentive suggesting it may be better to focus incentives on the “undecided” [27].

Two studies on cash transfers conducted following the introduction of COVID-19 vaccines showed a positive impact. In Sweden, Campos-Mercado et al. conducted a randomized control trial between May and July 2021 with over 8000 participants, offering 200 Swedish kroner (24 USD) for vaccination within 30 days of the vaccine becoming available to them [24]. This was associated with a 4.2 % increase in COVID-19 vaccination rates from the baseline of 71.6 %. A similar increase was reported for “intention” to get vaccinated. The effect was noted as similar across all socio-economic groups [24].

A non-randomized trial by Wong et al. using a difference-in-differences approach reported findings of a two-week pilot for a COVID-19 vaccination in four counties in North Carolina, USA [33]. A 25 USD cash card was given to adults who either received or drove someone to receive their first dose of COVID-19. Incentives were associated with a higher vaccine uptake rate. 41 % reported that the cash card was an important reason for vaccination, more so if Hispanic or other non-white and if from the lower-income groups. About 9 % reported they would not have been vaccinated if the cash card had not been offered, and 15 % waited to get vaccinated until they found an event that gave a cash card or other incentive [33].

### Non-cash transfers

We identified six studies on the effects of lottery entry on vaccine coverage, all assessing the impact at a population level, which showed limited to no effect. No studies were identified on the effects of other types of material incentives for increasing uptake of COVID-19 vaccines.

Studies by Barber and West and Sehgal et al. found modest improvements (approx. 1.5 % and 0.98 %, respectively) comparing vaccination rates in Ohio to a synthetic control from other states, weighted to match Ohio’s population (“synthetic Ohio”) [23], [30]. In Ohio, the Vax-A-Million campaign offered a ticket to a weekly prize of one million USD during five weeks in May-June 2021 for the residents that had received at least one dose of a COVID-19 vaccine. In absolute terms, between the lottery announcement and the end date, the increase in vaccination coverage was modest, from approximately 42 % to 47 % [30].

A cross-sectional study by Acharya et al. using a difference-in-differences analysis estimated an aggregate 2.1 % increase in vaccination coverage associated with lottery programs in the US (11 states with a program vs 28 states without) [22]. However, when analyzed separately by state, the results were mixed, with a positive association in some states but not in others [22].

Dave et al., also using a difference-in-differences method, compared states implementing incentives against those that did not, and found that lottery-based incentives in US states were not associated with a statistically significant effect on COVID-19 vaccination rates either before or after the announcement of the drawing [25]. A study by Law et al. comparing 15 states using lotteries with 31 non-lottery states did not find a significant effect when compared with the pre-lottery trend [28]. Results from Walkey et al. similarly found no difference when comparing first-dose vaccination rates in Ohio and with states without lotteries [28].

## Discussion

In this review, we explored cash and non-cash incentives offered for the adult population to improve vaccination coverage. While we found evidence of cash transfers increasing both the coverage and intention to be vaccinated, very few studies considered these effects at a population level and the ones that did found that the improvements were limited to a few percentage points in vaccination coverage. While the evidence is limited, findings on the experience with financial incentives and COVID-19 vaccines are largely consistent with findings from other adult vaccination such as hepatitis B, HPV, maternal tetanus, and influenza as well as the literature on the effectiveness of conditional cash transfers. According to evidence to date, lottery programs do not appear to have a meaningful impact on vaccination for COVID-19, with effects ranging from none to a 2.1 % increase in coverage, and no evidence was identified of positive effects of other non-cash incentives for COVID-19 or other adult vaccines. Further, most studies were conducted in the US with only four from low- and middle-income countries, none of which were on COVID-19.

Of note, for all vaccines, incentives were found to be more effective for the first dose than the second dose [26]. The reason behind this difference was not explored in any of the studies. There were also no studies identified evaluating the extent to which incentives would affect booster shots or other annual vaccination. As “point in time,” studies did not assess the extent to which payments “now” may affect health-seeking behavior in the future, including for vaccines that are offered without incentives. Only one study explored the impact of incentivizing one vaccination (HPV) and found no adverse impact vis-a-vis later vaccination for seasonal influenza [36]. Perhaps most surprising, there was no evidence presented in any of the studies on the extent to which incentives serve to address the concerns of those who are hesitant or even increase uptake among this specific subset of the population.

Data from other conditional cash transfer programs do show that when targeted at low-income populations, incentives can be highly cost-effective as well as lifesaving [48], [49], [50]. For COVID-19 vaccines, only one study reported on findings related to cost or marginal cost [22]. No analysis was identified on the extent to which programs were either cost-effective or cost-saving.

Some studies raise ethical concerns that financial incentives for vaccination could be construed as coercive [51], [52], and that in politically-divided contexts, government-promoted incentives might generate a backlash among those who are already hesitant, heightening suspicion of vaccination programs [51], [53]. Considering existing problems with vaccine hesitancy in many populations, this is an important concern to bear in mind and plan for. There is also a question about whether the use of financial incentives as a tool to promote vaccination uptake for COVID-19 may result in a societal expectation of these in the future, affecting both COVID-19 and other vaccination programs and one study did point out a delay based on an expectation of an incentive [33].

In terms of study design, the choice of the narrative review allowed us to conduct a comprehensive overview of the published literature. While we have borrowed some methods from the practice of a systematic review (e.g. PRISMA statement), because of the limited number of studies available and the breadth of scope of the research question, we were unable to conduct any grading of evidence or combined statistical data analysis that could help reduce bias in the data and conclusion [54]. With regard to limitations, our search focused on the adult population. Expanding it to routine or other childhood immunization could have yielded more results, particularly from low-and middle-income countries. The number of databases and languages used may have also limited the findings. There are also likely to be incentive schemes implemented in low-and middle-income countries but not described or investigated in peer-reviewed literature.

## Conclusion

Given the paucity of evidence, and the seriousness of the potential unintended consequences, it is remarkable how many governments, states, and cities offered incentives to increase vaccination coverage and did not embark on any type of implementation research to evaluate program effectiveness. Equally puzzling, while we may have evidence that some programs work, we do not necessarily understand why or for whom.

Moving forward, it will be important to monitor the impact of these incentive programs over time at both the individual and population levels. It will also be important to unpack the theory of change of these programs and understand whether their impact is more on those who are truly hesitant or those who are undecided. It will also be important to assess their cost-effectiveness against other known and effective ways to improve coverage, such as increasing trust with providers [55], increasing points of access and community outreach, and reducing out-of-pocket costs [56], [57], and providing clear information and reminders [58].

While we did not explore the role of provider-based incentives (“pay for performance”) for COVID-19 vaccines, these have shown promise to increase coverage for other types of adult vaccination and should be further explored [59], [60].

## CRediT authorship contribution statement

**Nina Schwalbe:** Conceptualization, Methodology, Data curation, Validation, Formal analysis, Wrote orignal draft, Reviewed, and Edited. **Layth Hanbali:** Data curation, Formal analysis and Contributed to the drafting, Revewing and Editing. **Marta C. Nunes:** Formal analysis, Writing, Review and Editing. **Susanna Lehtimaki:** Methodology, Formal analysis, Validation, Drafting, Reviewing and Editing.

## Ethical Approval

Ethical approval for this type of study is not required by our institute.

## Declaration of Competing Interest

The authors declare that they have no known competing financial interests or personal relationships that could have appeared to influence the work reported in this paper.

## Data Availability

No primary data were used for the research described in the article.
